# Benefits of Robot-Assisted Upper-Limb Rehabilitation from the Subacute Stage after a Stroke of Varying Severity: A Multicenter Randomized Controlled Trial

**DOI:** 10.3390/jcm13030808

**Published:** 2024-01-30

**Authors:** So Young Ahn, Soo-Kyung Bok, Ji Young Lee, Hyeon Woo Ryoo, Hoo Young Lee, Hye Jung Park, Hyun Mi Oh, Tae-Woo Kim

**Affiliations:** 1 Department of Rehabilitation Medicine, Chungnam National University Hospital, Daejeon 35015, Republic of Korea; 2 Department of Rehabilitation Medicine, College of Medicine, Chungnam National University Hospital, Daejeon 35015, Republic of Korea; 3 Department of Brain Injury Rehabilitation, National Traffic Injury Rehabilitation Hospital, Yangpyeong 12564, Republic of Koreapetitehj01@naver.com (H.J.P.); drcadaver@naver.com (T.-W.K.); 4 Department of Rehabilitation Medicine, Seoul National University Hospital, Seoul 03080, Republic of Korea

**Keywords:** robot-assisted therapy, stroke, rehabilitation, randomized controlled trial

## Abstract

Background: The aim of this study was to compare the clinical effectiveness of robot-assisted therapy with that of conventional occupational therapy according to the onset and severity of stroke. Methods: In this multicenter randomized controlled trial, stroke patients were randomized (1:1) to receive robot-assisted therapy or conventional occupational therapy. The robot-assisted training group received 30 min of robot-assisted therapy twice and 30 min of conventional occupational therapy daily, while the conventional therapy group received 90 min of occupational therapy. Therapy was conducted 5 days/week for 4 weeks. The primary outcome was the Wolf Motor Function Test (WMFT) score after 4 and 8 weeks of therapy. Results: Overall, 113 and 115 patients received robot-assisted and conventional therapy, respectively. The WMFT score after robot-assisted therapy was not significantly better than that after conventional therapy, but there were significant improvements in the Motricity Index (trunk) and the Fugl–Meyer Assessment. After robot-assisted therapy, wrist strength significantly improved in the subacute or moderate-severity group of stroke patients. Conclusions: Robot-assisted therapy improved the upper-limb functions and activities of daily living (ADL) performance as much as conventional occupational therapy. In particular, it showed signs of more therapeutic effectiveness in the subacute stage or moderate-severity group.

## 1. Introduction

Stroke is the most common cause of complex adult disabilities in high-income countries [1]. Each year, in the United States of America, 0.75 million people suffer from stroke, while the number of patients with stroke has been predicted to exceed 1.5 million by 2025 [2]. Upper-limb dysfunction after stroke is common, and upper-limb injuries persist even after three to six months in approximately 70–85% of cases, limiting the independence and social participation of affected individuals and thereby significantly reducing their quality of life. Hence, a crucial factor in stroke rehabilitation is to minimize the disability level and enhance the performance of the activities of daily living (ADL) by improving upper-limb functions [3].

In the last few years, the understanding of motor learning, neuroplasticity, and functional recovery after stroke has greatly expanded. Notably, functional recovery after stroke depends on neuroplasticity and brain reorganization. The brain is a highly dynamic system that can alter its neural circuits to display a spectrum of unique abilities. This brain plasticity can be mainly enhanced under a unique milieu of genetic, physiological, and structural events, as well as task-specific training for 1–3 months after ischemic stroke [4]. In particular, repetitive occupational training induces Hebbian plasticity by reinforcing simultaneously activated pathways [1], whereby high-dose intensive training and repetitive practice of specific functional tasks play effective roles [5].

Robot-assisted therapy is a novel approach to neurorehabilitation that is repetitive, focused, task-oriented, and quantifiable. Based on the previously described theoretical background, robot-assisted rehabilitation treatments have been increasingly applied to a wide array of neurological damage in the central nervous system. Various systematic reviews report that robot-assisted rehabilitation improves post-stroke ADL, arm function, and muscle strength [3,6,7]. In addition, for upper-limb rehabilitation, robots with an independent function evaluation and a periodic assessment of the training process to determine the training effects and areas that require complementation can enable more efficient operation of the training program.

The therapeutic effects of upper-limb rehabilitation robots have been reported in several Cochrane reviews [8,9]. According to the 2018 Cochrane review (45 trials, 1619 patients) [9], the reported effects of robot-assisted therapy in the rehabilitation of upper-limb functions, muscle strength, and performance of ADL were significantly higher than those of conventional therapy, with a high level of evidence. In several randomized controlled studies [10,11,12], the effects of robot-assisted therapy were favorable compared to conventional therapy, while robot-assisted therapy was reported to be more comfortable and safer for patients who performed the rehabilitation training more actively. Alternatively, some studies revealed that the use of upper-limb robots was not supported in routine clinical practice as the treatment of choice, and compared to conventional therapy, it did not lead to greater improvements in upper-limb functions or ADL performance [13].

While numerous studies have been conducted thus far regarding the effects of upper-limb robot-assisted therapy for stroke patients, its effects according to the onset and severity of stroke are unclear, and the benefits of robot-assisted therapy for the same frequency and duration as conventional therapy also remain unknown. Recently, neural signal monitoring, such as functional magnetic resonance imaging (fMRI), near-infrared spectroscopy (NIRS), and electroencephalography (EEG), has been applied to investigate the functional state and recovery in patients with stroke. Nevertheless, only a few studies have reported on the mechanism of neurological recovery, and no study has evaluated changes in brain functions in patients with stroke after upper-limb robot-assisted rehabilitation treatment [14].

Thus, this study aims to (1) compare robot-assisted therapy to conventional occupational therapy for the same frequency and duration in a randomized controlled trial among patients post-stroke; (2) quantitatively analyze variations in the effects of robot-assisted therapy across different subgroups of patients with stroke according to the onset and severity of stroke; and (3) identify the role of upper-limb robot-assisted therapy in the intervention and recovery mechanisms. The results are anticipated to contribute to strengthening the level of clinical evidence for the use of upper-limb robots in rehabilitation, based on which the clinical effectiveness of upper-limb robots in rehabilitation can be verified in a multicenter, large-scale study.

## 2. Materials and Methods

### 2.1. Participants

**Figure 1:**
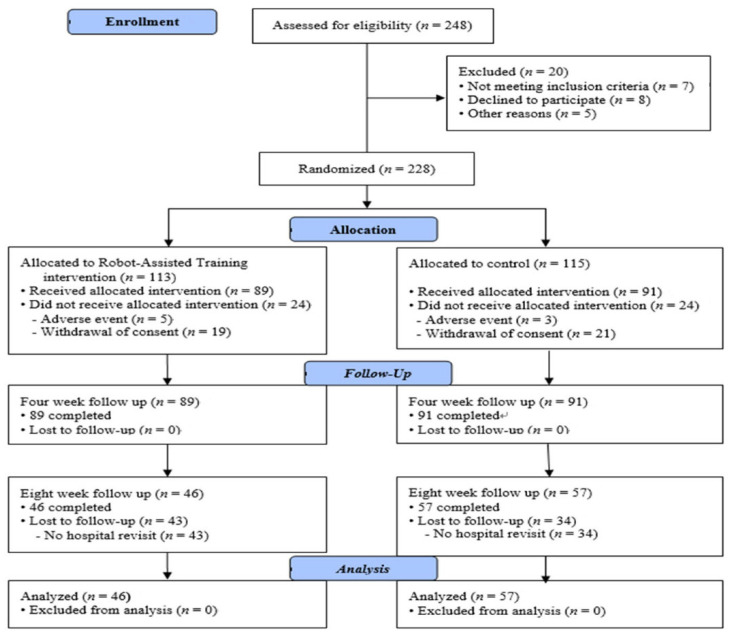
Flow diagram of the selection process in the study. RATG, robot-assisted training group. CG, control group.

From March 2019 to November 2021, we prospectively enrolled 228 individuals with upper-limb dysfunction after stroke who were admitted to two rehabilitation centers in South Korea. The inclusion criteria were as follows: (a) aged at least 20 years; (b) the first-ever stroke had occurred between 1 week and 2 years; (c) weakness of upper extremity (Medical Research Council Score 0–3); and (d) no severe cognitive function to allow cooperation (Mini-Mental State Examination Score ≥ 17). The exclusion criteria were as follows: (a) bilateral upper-extremity dysfunction due to quadriplegia or musculoskeletal problems; (b) severe spasticity (Modified Ashworth Scale Grades 3 and 4); and (c) inability to sit up unassisted. Patients were randomly assigned into two groups using a random number table: the robot-assisted training group (RATG) (*n* = 113), who underwent robot-assisted therapy combined with conventional therapy, and the control group (CG) (*n* = 115), who underwent conventional therapy only (Figure 1). This study was approved by the Institutional Review Board (IRB 2019-05-030) and was registered at the Clinical Research Information Service (Registration No: KCT0004132).

### 2.2. Standard Operating Protocol (SOP) Development

Through treatment and assessment, as well as the discussions of the advisory committee consisting of a rehabilitation medicine specialist, occupational therapists, and medical engineers, the protocol of conventional therapy was developed based on the methods used in clinical practice. In addition, therapists at each center were educated and trained according to the developed SOP for application in patient treatment.

#### 2.2.1. Conventional Occupational Therapy Protocol

The SOP was developed based on the scores of each item of the Fugl–Meyer Assessment (F-M) for upper-limb motion on the affected side. The treatment was performed according to the SOP (Supplementary File S1).

#### 2.2.2. InMotion ARM TM Protocol

The SOP was developed to incorporate the passive, active-assistive, and active modes.

Among the 5 result values obtained from running the adaptive mode 320 times in total (80 times per session × 4 sessions), the robot power data were used to determine, based on the level of active motion in the patients, how much resistance to apply when controlling the game mode for the Race/Slalom game (Supplementary File S2).

### 2.3. Study Design (and Procedure)

**Figure 2:**
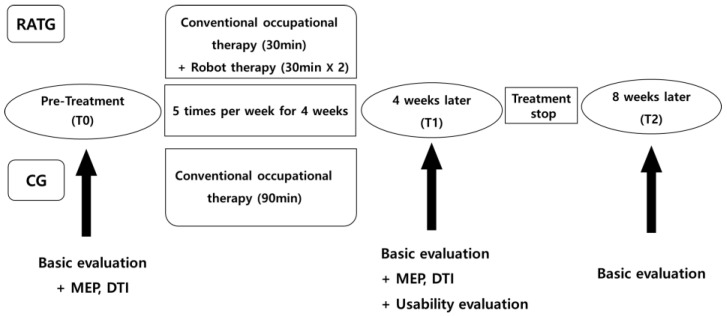
Flowchart of the study design. RATG, robot-assisted training group. CG, control group. MEP, motor-evoked potential. DTI, diffusion tensor imaging.

A prospective, randomized, controlled clinical trial was conducted. Unlike previous studies, in which the robot-assisted training group (RATG) received 30 min of training once a day, we used higher-intensity interventions, during which the RATG received 30 min of robot training twice a day and 30 min of conventional occupational therapy 5 days per week for 4 weeks. Participants in the control group (CG) received 90 min of conventional therapy with the same schedule as that used for the RATG. The assessments were performed at the beginning (T0) and end of the intervention (T1) and at 1-month follow-up (T2) (Figure 2).

### 2.4. Interventions or Robot-Assisted Training

The InMotion 2.0 Arm robot (Interactive Motion Technologies, Inc., Watertown, MA, USA), the commercial version of the MIT Manus, was used for the study. This device is a two-translational degrees-of-freedom planar robot that emphasizes shoulder and elbow movements in the horizontal plane, and the robot has a height-adjustable workstation. The participant was seated with the robot aligned with their midline and held the robot handle with the affected hand; forearm support was provided. The height of the robot arm was adjusted so the participant’s forearm could be parallel to the floor.

The active-assisted mode was mainly applied in this study. In this mode, the robot assisted the patients in reaching the targets when they could not perform the whole range of movement independently. The amount of assistance was adjusted and tuned based on the patient’s performance by using the robot’s built-in, performance-based control algorithm. According to the patient’s motor ability, other modes, such as passive, active, or resistive modes, could also be applied.

### 2.5. Assessment

#### 2.5.1. Primary Outcome

The primary outcome was upper-limb function improvement (defined using the Wolf Motor Function Test (WMFT) [15]).

#### 2.5.2. Secondary Outcomes

Secondary outcome measures included the Box and Block Test (BBT) [16], Fugl–Meyer Assessment (F-M) [17], Medical Research Council Score (MRC) [18], Motricity Index (MI) [19], hand grip strength, Modified Ashworth Scale (MAS) [20], the Korean version of the Modified Barthel Index (K-MBI) [21], Stroke Impact Scale 3.0 (SIS 3.0) [22], the Korean version of the Mini-Mental State Examination (K-MMSE) [23], Korea Geriatric Depression Scale (K-GDS) [24], EuroQol- 5 Dimension (EQ-5D) [25], robot built-in function evaluation, motor-evoked potential (MEP), and diffusion tensor imaging (DTI) findings.

#### 2.5.3. InMotion Robot Built-in Function Evaluation (Robot Kinematic Measure)

Through data collection, analysis, and quantitative measurement regarding the InMotion robot’s built-in function, the aiming, velocity, and smoothness of the motion and the improvement in the independent motion of the elbow and shoulder joints were evaluated. This study assessed four items: point-to-point movement test, circle-drawing movement test, playback static test, and round dynamic test.

#### 2.5.4. Assessment of Utility

The questionnaire on utility was developed to collect the basic data for using upper-limb robot-assisted rehabilitation in clinical settings. For this assessment, video calls, e-mails, and face-to-face consultations were mediated with experts, including the rehabilitation medicine specialists and occupational therapists at the centers currently using the InMotion^®^ ARM. For the secondary evaluation after the treatment, all participants and their therapists were requested to complete the questionnaire on the robot’s utility (Supplementary Files S3 and S4).

#### 2.5.5. Motor-Evoked Potential (MEP)

The MEP is a test used to objectively measure brain plasticity, which determines whether there is a conduction abnormality in the pathways from the cerebral motor cortex to peripheral motor neurons by measuring changes in latency and amplitudes. At 120% and 140% intensity of the resting motor threshold (RMT), the hot spot was stimulated ten times, and the mean values of latency and amplitude (peak-to-peak amplitude) were estimated (acceptable at latency < 25 and prolonged at latency > 25). The resting motor threshold, defined as the lowest stimulus intensity able to evoke 5 of 10 MEPs with an amplitude of at least 50 mV, was determined by holding the stimulation coil over the optimal scalp position, defined as the position from which MEPs with maximal amplitude were recorded for FDI muscle [26].

#### 2.5.6. Diffusion Tensor Imaging (DTI)

The DTI is a test used to evaluate brain plasticity, for which the neural pathways of the corticospinal tract (CST) are analyzed. The corticospinal tract was reconstructed using two regions of interest (ROIs). The seed ROI was set to the area of the pontomedullary junction, and the target ROI was set to the area of the anterior CST of mid-pons. The mean diffusivity, fractional anisotropy (FA), FA ratio (affected side vs. healthy side), and tract volume were estimated. The proportion of FA was calculated by dividing the value of the CST in the affected hemisphere by the value of the CST in the unaffected hemisphere. The person who analyzed DTI data was blinded to those collecting clinical data [27].

### 2.6. Statistical Analyses

All data were analyzed using SAS statistical software (Institute Inc., Cary, NC, USA; Version 9.4). Continuous variables are expressed as mean ± standard deviation, and categorical variables are expressed as a count (%). Baseline descriptive statistics were compared using the Mann–Whitney test/independent *t*-test for continuous data and the chi-square test for categorical data. The Wilcoxon signed-rank test/paired *t*-test was used for comparisons within groups both at baseline and after treatment. The Mann–Whitney test was used to compare the differences in values between groups before and after the intervention. For all analyses, statistical significance was set at *p* < 0.05.

## 3. Results

### 3.1. Participants’ Baseline Characteristics

The study involved two rehabilitation centers in South Korea from March 2019 to November 2021, resulting in 228 patients with stroke, with 113 patients in the RATG and 115 in the CG.

Analysis was performed for 89 patients in the RATG and 91 patients in the CG.

The mean age of the patients was 59.2 years, and the number of patients evaluated less than three months after stroke onset was 120 (66.7%). In the subacute stage, the mean stroke duration in the treatment group was 43.5 ± 15.2 days, and in the control group, it was 51.4 ± 18.4 days. In the chronic stage, the mean stroke duration was 295.7 ± 105.4 days in the treatment group and 320.8 ± 118.9 days in the control group.

No significant differences were found in the baseline characteristics of the participants between the two groups (Table 1).

### 3.2. Comparisons of Robot-Assisted Therapy with Conventional Therapy (T0–T1)

In terms of the primary outcome, all groups showed significant improvement after the treatment, but the degree of improvement did not differ significantly between the intervention groups (Table 2). In terms of the secondary outcomes, the comparison between before and after treatment for each group revealed a significant improvement across all clinical evaluations except the MAS and K-GDS. The between-group comparison of the pre-/post-treatment variation demonstrated significant improvement in F-M (*p* = 0.0394) and MI (trunk) (*p* = 0.0005) for the RATG compared with that for the control group. In the robot built-in function evaluation, the RATG showed improvements compared to the control group in smoothness and maximal velocity in the point-to-point movement test, and independence in the circle-drawing movement test.

In the neurophysiological test using the MEP, no significant improvement was observed in the within-group and between-group comparisons of the pre-/post-treatment variation (Table 2).

### 3.3. Comparisons of Robot-Assisted Therapy with Conventional Therapy (T0–T2)

Significant improvement was observed across all clinical evaluations except the MAS and K-GDS. The RATG showed a significant improvement in the MI (trunk).

In the robot built-in function evaluation, the previously detected pattern of significant improvement in the RATG in the pre-/post-treatment analysis was not observed.

### 3.4. Subgroup Analysis Depending on the Severity

Based on the evidence provided by Elizabeth et al. (2017) [28] and the F-M, scores of 0–28, 29–42, and 43–66 were defined as the severe, moderate, and mild groups, respectively.

Regarding the primary outcome, across all levels of severity, a significant improvement was observed after treatment in both the CG and RATG, but no significant variation was observed among the intervention groups.

Considering the secondary outcomes, for the BBT, F-M, and K-MBI, both the CG and RATG showed a significant improvement after the treatment across all levels of severity, but no significant variation was found among the intervention groups.

For MRC, the level of significant improvement was greater in the severe stroke group than in the mild and moderate stroke groups for both the CG and RATG. A significant improvement in wrist flex–extension muscle strength in the moderate group was observed for the RATG. For the neurophysiological test using the MEP and DTI, the pre-/post-treatment test of each intervention group and the between-group comparison indicated no significant variation (Table 3).

### 3.5. Subgroup Analysis between Subacute and Chronic Patients

Depending on the time of neurological recovery, the subacute phase was defined as 1 week to 3 months, and the chronic phase was defined as 3 months to 2 years [29].

Regarding the primary outcome, all groups significantly improved after the treatment, but no significant variation was observed among intervention groups.

In terms of the secondary outcomes, for the F-M, MI (upper and lower limbs), and hand grip strength, all groups showed a significant improvement after the treatment, but no significant variation was observed among the intervention groups.

For the MI (trunk), a significant improvement was observed in only the subacute group for the RATG.

For the MRC, the level of significant improvement was greater in the subacute group than in the chronic group in most cases, while a significant improvement in wrist extension was observed in the subacute group for the RATG.

In the robot built-in function evaluation, the subacute group showed a significant improvement across all tested items after the robot-assisted treatment, and a significant variation among the intervention groups was observed in eight out of the nine tested items. While conventional therapy did not significantly improve the chronic group, robot-assisted therapy significantly improved in six out of the nine tested items, three of which displayed a significant variation among the intervention groups.

Considering the DTI and MEP, the chronic group showed a significant improvement after the robot-assisted treatment only for the mean FA in DTI (*p* = 0.0348) (Table 4).

## 4. Discussion

The upper-limb robot-assisted rehabilitation using InMotion 2.0 in this study led to significant improvement in most tests, except for the MAS and K-GDS, indicating that robot-assisted therapy for upper-limb rehabilitation was as effective as conventional occupational therapy in the clinical improvement in upper-limb functions and ADL performance. Furthermore, in addition to the comparison between robot-assisted therapy and conventional therapy for upper-limb rehabilitation, we also performed a subgroup analysis based on the stroke onset and severity due to the predicted positive role of robot-assisted therapy in the treatment of a group of patients in accordance with post-stroke duration and stroke severity, which can help in developing treatment plans for patients with stroke.

Robot-assisted therapy is an innovative approach to post-stroke neurological rehabilitation that comprises intensive, repetitive, interactive, and individualized practice. Various types of upper-limb rehabilitation robots are currently used, and InMotion is the most widely used robot in robot-assisted training for post-stroke patients with moderate-to-severe upper-limb damage. Successful results were reported using the InMotion test in a clinical study involving over 900 patients with stroke, and approximately 250 InMotion robots are currently used worldwide [1]. According to the systematic review by Ferreira et al. [7], the MIT-Manus robot-assisted therapy led to a significant improvement in F-M and WMFT scores compared to those after conventional therapy for rehabilitation. However, the study by Rodgers et al. [13] reported that the MIT-Manus robot-assisted therapy did not lead to a greater level of improvement in upper-limb functions or ADL performance than conventional therapy. In this study, similarly, the lack of significant improvement in WMFT scores after the robot-assisted therapy was a limitation, given the fact that the WMFT score was used to determine the level of improvement in upper-limb functions as the primary outcome.

For the MRC in this study’s assessment of muscle strength, a significant post-treatment improvement was mostly observed in the subacute group compared to the chronic group. In the analysis according to severity, a significant post-treatment improvement was observed in the severe group, consistent with several previous studies [3,11,30].

The subgroup analysis according to severity also indicated that the moderate group exhibited a significant improvement in wrist flexion–extension MRC after robot-assisted therapy compared to the RATG and the control group. The ΔMRC was −0.12 ± 0.60 for the mild group, indicating a limitation due to the ceiling effect of the MRC evaluation, while the strongest effect in the moderate group is presumed to be due to the task difficulty in the severe group.

Alternatively, the subgroup analysis according to onset revealed that the subacute group exhibited a significant improvement in wrist extension after robot-assisted therapy. InMotion is a shoulder–elbow unit for planar movement and an end-effector-based robot with two degrees of freedom, whose use in robot-assisted therapy requires splint support for the forearm due to the lack of control on the affected arm. The systematic review by Prange et al. [31] reported an improvement in the muscle strength and functions of the shoulder and elbow after InMotion-based treatment. In this study, likewise, the muscle strength showed an improvement for the shoulder, elbow, and wrist in both the RATG and CG. On the other hand, the significant improvement in the wrist muscle strength in the RATG was contradictory to the observations of Prange et al. [31]. This was attributed to the wrist muscle isometric contraction through the use of InMotion, and it is anticipated that the improvement in the wrist muscle strength would increase the level of improvement in upper-limb functions on the affected side.

In addition, a significant improvement in the MI (trunk) after the robot-assisted therapy was observed in the follow-up evaluation and the analysis according to onset for the subacute group, compared to the control group. It is presumed that, as the patient sits with the midline in alignment with the robot in robot-assisted therapy, while the height of the robot is adjusted so that the patient’s forearm is in parallel with the robot, the core stability and trunk muscle synergy can improve through an increased level of intensive training and task performance. This is consistent with observations by Carpinella et al. [10], indicating an improvement in shoulder–elbow coordination and a substantial decrease in abnormal trunk movements after robot-assisted upper-limb rehabilitation.

In this study, the MEP and DTI facilitated the objective analyses of changes in the chronic stage, making these findings novel, as no study has examined neurophysiological changes in patients with stroke following robot-assisted therapy, and previous studies applied conventional therapy. However, contrary to expectations, there were changes in MEP and DTI in the chronic phase, and a clear answer was not obtained even after reviewing other papers. This remained a limitation of this study. Regarding previous research on MEP among patients with stroke, the sole report is of an improvement in the clinically favorable prognosis in robot-assisted upper-limb rehabilitation in the presence rather than the absence of MEP [10]. Electroencephalography (EEG) is also suggested to assess the recovery of motor function, through brain symmetry, spectral power, and event-related measures [32].

While the pre-/post-treatment test results and the variations among the intervention groups in the follow-up and severity-based subgroup analyses did not exhibit statistical significance, the onset time analysis revealed a significant increase in the mean FA in DTI and amplitude in MEP in the chronic group for the RATG. While there is a correlation between FA in DTI and white matter integrity, a decrease in FA reflects axonal loss and Wallerian degeneration [33]. Wallerian degeneration has been widely examined across patients with chronic stroke, and for the period of several months to several years after stroke, FA decreases along the pyramidal tract on the affected side [34]. An increase in FA after conventional therapy for rehabilitation is correlated with improvement in F-M-UE [35], and according to the study by Mrachacz-kersting et al., an increase in amplitude in MEP after conventional therapy for rehabilitation indicated a functional improvement in chronic patients with stroke [36]. In addition, Volz et al. [37] reported motor impairment in chronic patients with stroke based on decreases in ipsilesional motor cortex excitability (73%), interhemispheric connectivity (64%), and structural CST damage (51%). Hara [5] reported that post-stroke recovery is induced by the facilitation of motor-related areas on the non-affected side in the acute phase, whereas in the chronic phase, it is induced by an increase in the cortex activity in motor-related areas on the affected side, lending additional support for the results in this study.

It is a well-accepted fact that a repetitive and intensive exercise regime leads to better performance in stroke patients as well as a clinical and radiological enhancement in neural plasticity [5,11,27,38]. In the subgroup analysis conducted in this study according to stroke severity and onset, the mechanism through which robot-assisted therapy was more effective than conventional treatment was not revealed, but upper-extremity function has been found to improve in patients in the moderate and subacute stages of stroke. This suggests that robot-assisted therapy plays an effective role in providing a large number of intensive training exercises and repetitive tasks that cannot be performed in conventional therapy.

Furthermore, a standard clinical protocol was developed in this study for the use of InMotion in robot-assisted upper-limb rehabilitation as well as conventional occupational therapy, as there was no SOP or established protocol for the use of upper-limb rehabilitation robots in patients with stroke, and no association was observed with changes in brain neural tracts. The findings of this study are anticipated to provide valuable basic data in the clinical application of upper-limb rehabilitation robots.

### Limitations

The strength of this study is that it is a multicenter, large-scale randomized controlled trial, but there are several limitations. First, the number of chronic patients (*n* = 60) was only half the number of subacute patients (*n* = 120), and in the follow-up evaluation, only 103 out of the 180 patients completed the follow-up a month after the completion of the treatment, with the loss of 77 patients.

Second, the wrist muscle strength showed a significant improvement in the moderate and subacute groups compared to that in the control group. Regrettably, the wrist could not be independently analyzed among the three subcategories of the F-M-UE to represent the upper-limb functional recovery. The DTI and MEP improved in the chronic group after robot-assisted therapy. The limitations are the lack of significant improvement in other tests reflecting functional and muscle strength improvements and a clear explanation for the improvements in the neurophysiological test. Further studies are needed to investigate the effects of robot-assisted therapy, which should involve a greater number of patients with chronic stroke and a long-term follow-up, with a focus on kinematic analysis to provide an account of the recovery of upper-limb motor functions. In addition, considering the similar effects of robot-assisted therapy and conventional therapy, studies should also investigate the cost-effectiveness of less labor-intensive robot-assisted therapy, especially in terms of labor cost.

## 5. Conclusions

The findings in this study suggested that the clinical effects of upper-limb robot-assisted rehabilitation on the improvement in upper-limb functions and ADL performance were as desirable as conventional occupational therapy. In a subgroup analysis of onset time and severity, upper-limb robot-assisted therapy improved trunk control and was more effective in the subacute stage or patients with moderate-severity stroke. In addition, the analysis of the effects of upper-limb robot-assisted rehabilitation based on the clinical type of stroke patients indicated a directionality for the successful selection of rehabilitation target patients and effective robot-assisted treatment. The results of this study are anticipated to serve as the basic data for future follow-up studies and provide evidence for the clinical application of upper-limb rehabilitation robots. The data will also prove valuable in promoting the robot industry through collaborations across relevant institutions, robot developers and researchers, and rehabilitation robot companies.

## Figures and Tables

**Table 1:** Demographic and clinical characteristics of patients.

	Control (*n* = 89)	Treatment (*n* = 91)	*p*-Value
Age, mean ± SD, y	60.1 ± 13.9	58.4 ± 14.9	0.432
Sex: male, No. (%)			
Male	56 (62.9)	62 (68.1)	0.531
Female	33 (37.1)	29 (31.8)	
Side of lesion, No. (%)			
Lt.	52 (58.4)	48 (52.7)	0.443
Rt.	37 (41.6)	43 (47.3)	
Type of stroke, No. (%)			
ICH	27 (30.3)	30 (33.0)	0.811
SAH	2 (2.3)	1 (1.1)	
Infarction	50 (67.4)	60 (65.9)	
Onset, No. (%)			
Subacute	56 (62.9)	64 (70.3)	0.344
Chronic	33 (37.1)	27 (29.7)	
Severity, No. (%)			
Mild	28 (31.5)	17 (18.7)	0.492
Moderate	11 (12.3)	10 (11)	
Severe	50 (56.2)	64 (70.3)	
MMSE	22.13	21.70	0.764

*p*-Value: *t*-test (continuous variables); chi-square test or Fisher’s exact test (categorical variables); SD, standard deviation. ICH, intracerebral hemorrhage. SAH, subarachnoid hemorrhage. MMSE, Mini-Mental State Examination.

**Table 2:** Between-group comparison of the pre-/post-treatment.

	Changes = Baseline–Post-Treatment		*p*-Value
	Control (*n* = 89)	Treatment (*n* = 91)	
Primary Outcome			
WMFT	−6.00 ± 10.20	−9.09 ± 14.60	0.176
Secondary Outcome			
F-M	−3.45 ± 12.37	−7.77 ± 11.46	0.039 *
MI (trunk)	−6.58 ± 17.05	−14.15 ± 20.39	<0.001 *
MEP			
CMAP latency (ms)	−0.43 ± 16.93	−2.78 ± 22.54	0.909
CMAP amplitude (mV)	0.17 ± 18.19	0.05 ± 23.14	0.663
MEP latency (ms)	−1.08 ± 10.78	−0.92 ± 15.73	0.783
MEP amplitude (mV) 120	−0.07 ± 0.76	1.32 ± 11.63	0.759
MEP amplitude (mV) 140	−0.10 ± 0.88	1.31 ± 11.66	0.744
MEP 140/120	−0.42 ± 1.16	1.22 ± 11.69	0.648
Diffusion Tensor Imaging			
Number of tracts	6197.44 ± 51,253.92	−556.07 ± 8273.53	0.657
FA mean	0.01 ± 0.09	−0.01 ± 0.14	0.754
FA ratio	−0.02 ± 0.14	−0.03 ± 0.32	0.630
Robot Built-In Function Evaluation (Robot Kinematic Measure)			
Point-to-point movement test (smoothness)	0.00 ± 0.11	−0.03 ± 0.09	0.031 *
Point-to-point movement test (reach error)	0.02 ± 0.04	0.05 ± 0.13	0.075
Point-to-point movement test (mean velocity)	−0.02 ± 0.10	−0.03 ± 0.04	0.503
Point-to-point movement test (maximal velocity)	−0.01 ± 0.11	−0.04 ± 0.08	<0.001 *
Point-to-point movement test (path error)	0.01 ± 0.02	0.01 ± 0.02	0.589
Circle-drawing movement test (circle size)	−0.01 ± 0.08	0.00 ± 0.12	0.832
Circle-drawing movement test (independence)	−0.05 ± 0.18	−0.16 ± 0.21	<0.001 *

Values are presented as mean ± standard deviation; *p*-value: independent *t*-test; * = statistically significant (*p* < 0.05). WMFT, Wolf Motor Function Test. F-M, Fugl–Meyer Assessment. MI, Motricity Index. CMAP, compound muscle action potential. MEP, motor-evoked potential. FA, fractional anisotropy.

**Table 3:** Between-group comparison of the pre-/post-treatment based on the severity.

		Change = Baseline–Post-Treatment		Mean ± SD	*p*-Value ^1^	*p*-Value ^2^
WMFT		Mild (*n* = 45)	Control (*n* = 28)	−2.93 ± 6.05	0.002 *	0.383
			Treatment (*n* = 17)	−4.59 ± 11.72	0.046 *	
		Moderate (*n* = 21)	Control (*n* = 11)	42.70 ± 20.96	0.012 *	0.463
			Treatment (*n* = 10)	−11.10 ± 13.92	0.045 *	
		Severe (*n* = 114)	Control (*n* = 50)	−7.56 ± 11.43	<0.001 *	0.409
			Treatment (*n* = 64)	−9.97 ± 15.34	<0.001 *	
MRC	Elbow Flexion	Mild (*n* = 45)	Control (*n* = 28)	−0.18 ± 0.61	0.234	0.637
			Treatment (*n* = 17)	−0.12 ± 0.60	0.688	
		Moderate (*n* = 21)	Control (*n* = 11)	−0.20 ± 0.63	0.625	0.222
			Treatment (*n* = 10)	−0.70 ± 0.82	0.063	
		Severe (*n* = 114)	Control (*n* = 50)	−0.42 ± 0.70	<0.001 *	0.979
			Treatment (*n* = 64)	−0.42 ± 0.92	<0.001 *	
	Elbow Extension	Mild (*n* = 45)	Control (*n* = 28)	−0.18 ± 0.61	0.234	0.637
			Treatment (*n* = 17)	−0.12 ± 0.60	0.688	
		Moderate (*n* = 21)	Control (*n* = 11)	−0.20 ± 0.63	0.625	0.377
			Treatment (*n* = 10)	−0.60 ± 0.84	0.125	
		Severe (*n* = 114)	Control (*n* = 50)	−0.40 ± 0.78	0.001 *	0.748
			Treatment (*n* = 64)	−0.38 ± 1.02	<0.001 *	
	Wrist Flexion	Mild (*n* = 45)	Control (*n* = 28)	−0.18 ± 0.67	0.273	0.827
			Treatment (*n* = 17)	−0.29 ± 0.69	0.188	
		Moderate (*n* = 21)	Control (*n* = 11)	−0.30 ± 0.48	0.250	0.022 *
			Treatment (*n* = 10)	−1.20 ± 0.79	0.008 *	
		Severe (*n* = 114)	Control (*n* = 50)	−0.28 ± 0.61	0.003 *	0.526
			Treatment (*n* = 64)	−0.20 ± 1.01	0.075	
	Wrist Extension	Mild (*n* = 45)	Control (*n* = 28)	−0.14 ± 0.65	0.398	0.664
			Treatment (*n* = 17)	−0.29 ± 0.69	0.188	
		Moderate (*n* = 21)	Control (*n* = 11)	−0.30 ± 0.67	0.500	0.049 *
			Treatment (*n* = 10)	−1.10 ± 0.88	0.016 *	
		Severe (*n* = 114)	Control (*n* = 50)	−0.22 ± 0.62	0.023 *	0.697
			Treatment (*n* = 64)	−0.27 ± 0.98	0.010 *	

Values are presented as mean ± standard deviation; *p*-value ^1^: Wilcoxon signed-rank test, comparison before and after treatment by group; *p*-value ^2^: Mann–Whitney U test, between-group comparison of the pre-/post-treatment variation; * = statistically significant (*p* < 0.05). WMFT, Wolf Motor Function Test. MRC, Medical Research Council Score.

**Table 4:** Between-group comparisons of the pre-/post-treatment as onset time.

		Change = Baseline–Post-Treatment		Mean ± SD	*p*-Value ^1^	*p*-Value ^2^
WMFT		Subacute (*n* = 120)	Control (*n* = 56)	−8.07 ± 10.48	<0.001 *	0.296
			Treatment (*n* = 64)	−11.53 ± 16.22	<0.001 *	
		Chronic (*n* = 60)	Control (*n* = 33)	−2.48 ± 8.76	0.039 *	0.182
			Treatment (*n* = 27)	−3.30 ± 7.15	0.007 *	
MRC	Wrist Flexion	Subacute (*n* = 120)	Control (*n* = 56)	−0.30 ± 0.71	0.002 *	0.09
			Treatment (*n* = 64)	−0.61 ± 0.88	<0.001 *	
		Chronic (*n* = 60)	Control (*n* = 33)	−0.18 ± 0.39	0.031 *	0.002 *
			Treatment (*n* = 27)	0.33 ± 0.88	0.063	
	Wrist Extension	Subacute (*n* = 120)	Control (*n* = 56)	−0.25 ± 0.74	0.022 *	0.045 *
			Treatment (*n* = 64)	−0.59 ± 0.87	<0.001 *	
		Chronic (*n* = 60)	Control (*n* = 33)	−0.15 ± 0.36	0.063	0.021 *
			Treatment (*n* = 27)	0.19 ± 0.92	0.500	
MI	(ARM + LEG)/2	Subacute (*n* = 120)	Control (*n* = 56)	−11.10 ± 13.66	<0.001 *	0.795
			Treatment (*n* = 64)	−11.79 ± 13.77	<0.001 *	
		Chronic (*n* = 60)	Control (*n* = 33)	−3.62 ± 7.33	0.007 *	0.649
			Treatment (*n* = 27)	−2.02 ± 4.73	0.006 *	
	trunk	Subacute (*n* = 120)	Control (*n* = 56)	−7.09 ± 15.22	0.001 *	0.002 *
			Treatment (*n* = 64)	−18.47 ± 21.74	<0.001 *	
		Chronic (*n* = 60)	Control (*n* = 33)	−5.73 ± 20.00	0.203	0.869
			Treatment (*n* = 27)	−3.93 ± 11.84	0.125	
MEP	MEP amplitude (mV) 120	Subacute (*n* = 120)	Control (*n* = 56)	−0.07 ± 0.90	0.141	0.700
			Treatment (*n* = 64)	−0.08 ± 0.20	0.001 *	
		Chronic (*n* = 60)	Control (*n* = 33)	0.05 ± 0.19	0.054	0.004 *
			Treatment (*n* = 27)	5.37 ± 22.92	0.109	
DTI	FA mean	Subacute (*n* = 120)	Control (*n* = 56)	0.00 ± 0.04	0.903	0.474
			Treatment (*n* = 64)	0.01 ± 0.13	0.522	
		Chronic (*n* = 60)	Control (*n* = 33)	0.02 ± 0.16	0.379	0.039 *
			Treatment (*n* = 27)	−0.06 ± 0.13	0.044 *	

Values are presented as mean ± standard deviation; *p*-value ^1^: Wilcoxon signed-rank test, comparison before and after treatment by group; *p*-value ^2^: Mann–Whitney U test, between-group comparison of the pre-/post-treatment variation; * = statistically significant (*p* < 0.05). WMFT, Wolf Motor Function Test. MRC, Medical Research Council Score. MI, Motricity Index. MEP, motor-evoked potential. DTI, diffusion tensor imaging. FA, fractional anisotropy.

## Data Availability

The original contributions presented in the study are included in the article/Supplementary Materials, further inquiries can be directed to the corresponding author.

## Supplementary Materials

The following supporting information can be downloaded at: https://www.mdpi.com/article/10.3390/jcm13030808/s1, Supplementary File S1, File S2: based on the level of active motion in the patients the robot power data were used to determine how much resistance to apply when controlling the game mode for the Race/Slalom game; File S3, File S4: Robot assisted rehabilitation after stroke.
