# Supplementary material for: Benefits of Robot-Assisted Upper-Limb Rehabilitation from the Subacute Stage after a Stroke of Varying Severity: A Multicenter Randomized Controlled Trial
Source: J Clin Med. 2024 Jan 30;13(3):808. doi: 10.3390/jcm13030808 (PMC10856364; doi:10.3390/jcm13030808)
Supplement: Supplementary file 1 [file jcm-13-00808-s001.zip › jcm-2830640-supplementary.pdf]

Journal of
Clinical Medicine

MDPI

Supplementary Materials

File S1.

**F-M (Fugl-Meyer Assessment)**		**Conventional Therapy**	**Training**
Item A on the affected side	≤18	Shoulder girdle strength (Assisted task performance)<br>Rotator cuff strength (Assisted task performance)<br>Reaching pattern training (Assisted task performance)	-ROM arc for shoulder stability training<br>-strength increasing<br>:sending, climbing board<br>-pattern training using Grahamizer
	≥19	Shoulder girdle strength (Active task performance)<br>Rotator cuff strength (Active task performance)<br>Reaching pattern training (Active task performance)	
Item B on the affected side	≤5	Wrist stability strength (Assisted task performance)<br>Wrist extensor muscle strength (Assisted task performance)	-wrist stability strength using cup board<br>-cuff weight, resistance exercise using dumbbell exercise<br>-pronation, supination activity for wrist stability and strength ex) cue card.
	≥6	Wrist stability strength (Active task performance)<br>Wrist extensor muscle strength (Active task performance)	
Item C on the affected side	≤7	Hand intrinsic muscle strength (Assisted task performance)<br>Hand grasp pattern strength (Assisted task performance)<br>Hand grasp pattern training (Assisted task performance)	-hand motion training<br>pinch pin, pegboard, grasp pattern training using pin board<br>-change of grade level in performing the motion on the affected side to enhance the strength<br>:putty, digit-flex, finger weight or cuff weight
	≥8	Hand intrinsic muscle strength (Active task performance)<br>Hand grasp pattern strength (Active task performance)<br>Hand grasp pattern training (Active task performance)	
Item D on the affected side	≤3	Repetitive reaching & grasp stability increase training (Assisted task performance)	-coordination motion increase training<br>:stacking cone, horizontal/vertical ring tree task performance
	≥4	Repetitive reaching & grasp pattern training (Active task performance)	

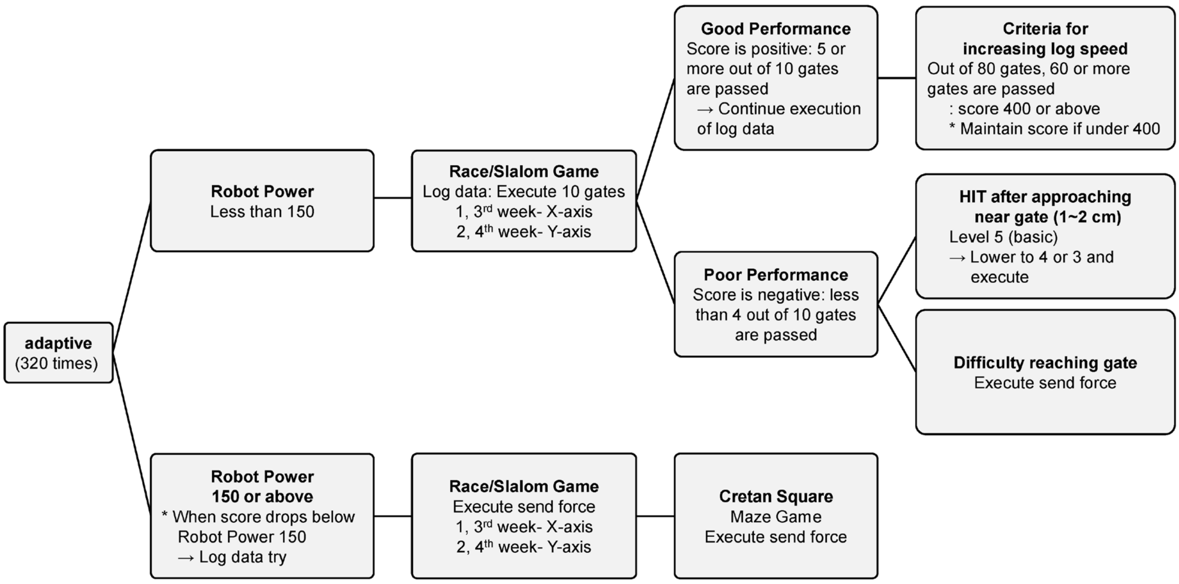

File S2. Based on the level of active motion in the patients the robot power data were used to determine how much resistance to apply when controlling the game mode for the Race/Slalom game.

File S3.

Question	Highly Dissatisfied	Dissatisfied	Moderate	Satisfied	Highly Satisfied
1. Is the preparation stage (taking the seat and wearing the robot) prior to using the upper limb robot comfortable?	①	②	③	④	⑤
2. Is the holding of the upper limb robot in its fixed state comfortable?	①	②	③	④	⑤
3. Does the process of rehabilitation using the upper limb robot feel comfortable?	①	②	③	④	⑤
4. Does the process of rehabilitation using the upper limb robot feel safe?	①	②	③	④	⑤
5. Is the treatment result easily accessible after the rehabilitation using the upper limb robot?	①	②	③	④	⑤
6. Is the overall experience of the robot-assisted rehabilitation therapy satisfactory?	①	②	③	④	⑤
7. Do you feel that the upper limb movements have improved after the treatment using the upper limb robot compared to the time before the treatment?	①	②	③	④	⑤
8. Do you intend to apply the treatment using the upper limb robot again in future?	①	②	③	④	⑤

File S4. Robot assisted rehabilitation after stroke.

Question	Strongly Disagree	Disagree	Moderate	Agree	strongly Agree
1. I'd like the upper limb robot to be used more frequently in treatments.	①	②	③	④	⑤
2. I feel the upper limb robot exhibits more complexity than is required.	①	②	③	④	⑤
3. I feel the upper limb robot is structured for convenient use.	①	②	③	④	⑤
4. I feel technical support is necessary for the use of the upper limb robot.	①	②	③	④	⑤
5. I feel the varying functions of the upper limb robot are in harmony.	①	②	③	④	⑤
6. I think the composition of the upper limb robot system is highly inconsistent.	①	②	③	④	⑤
7. I think most therapists would learn how to operate the upper limb robot rapidly.	①	②	③	④	⑤
8. I think the process of using the upper limb robot is highly complex and inconvenient.	①	②	③	④	⑤
9. I am highly confident regarding the use of the upper limb robot.	①	②	③	④	⑤
10. I had to learn many things prior to the use of the upper limb robot.	①	②	③	④	⑤
